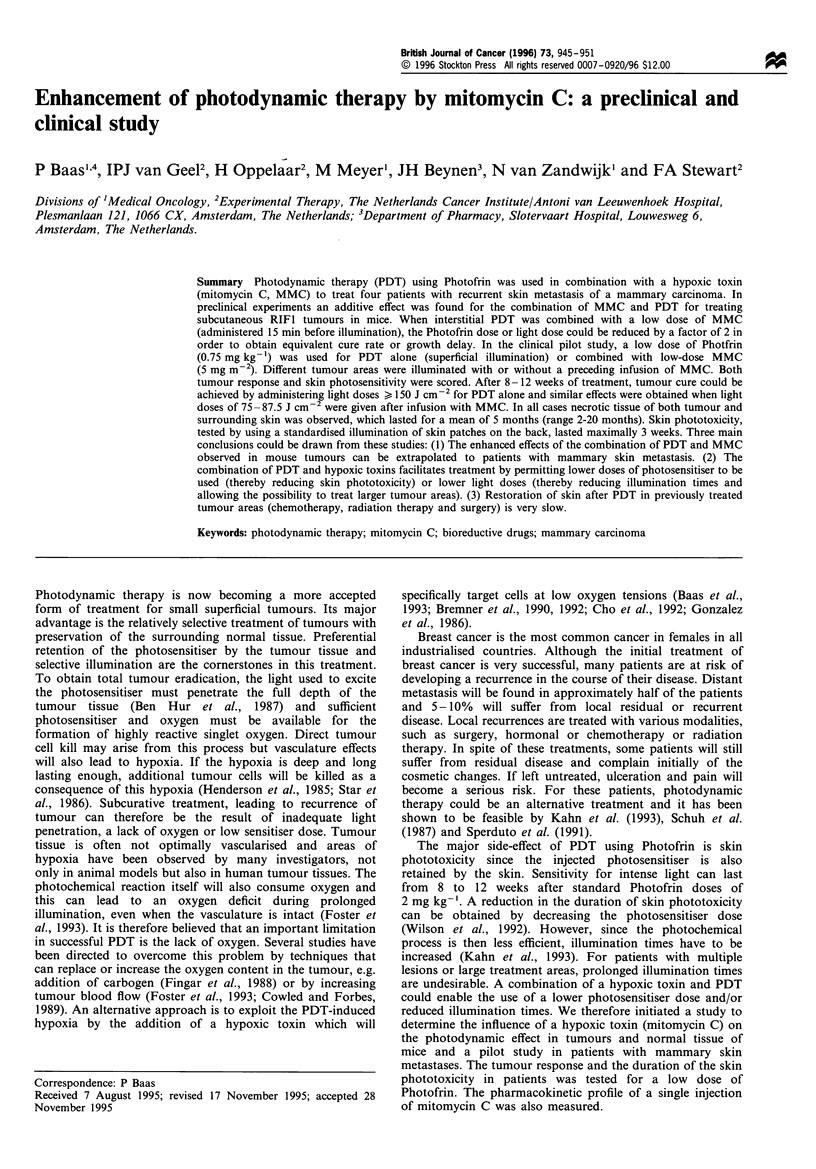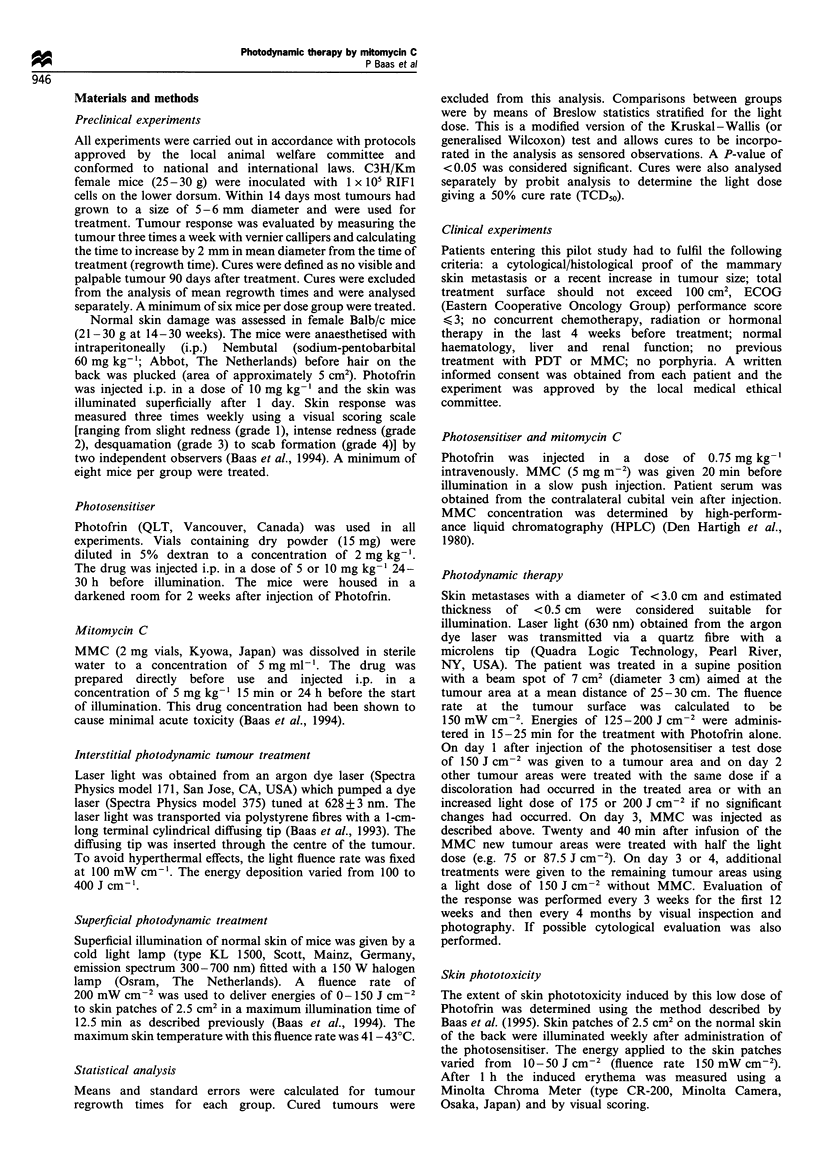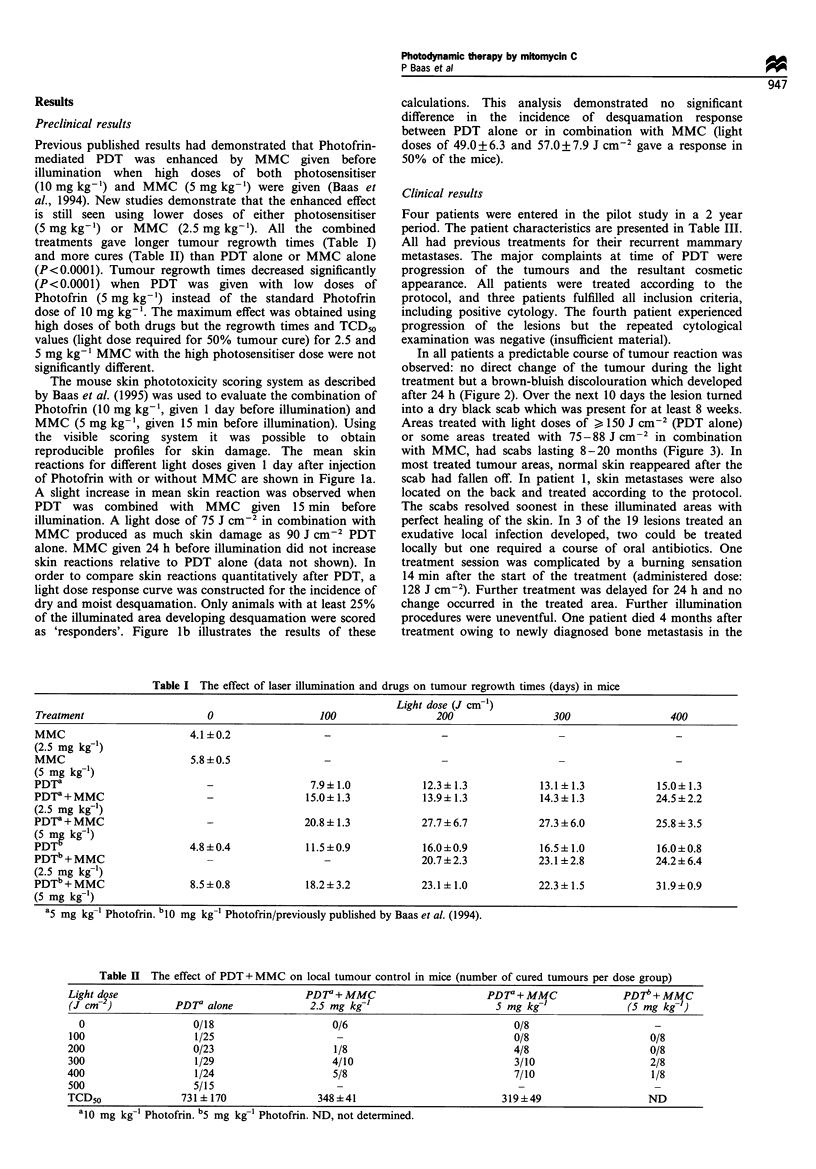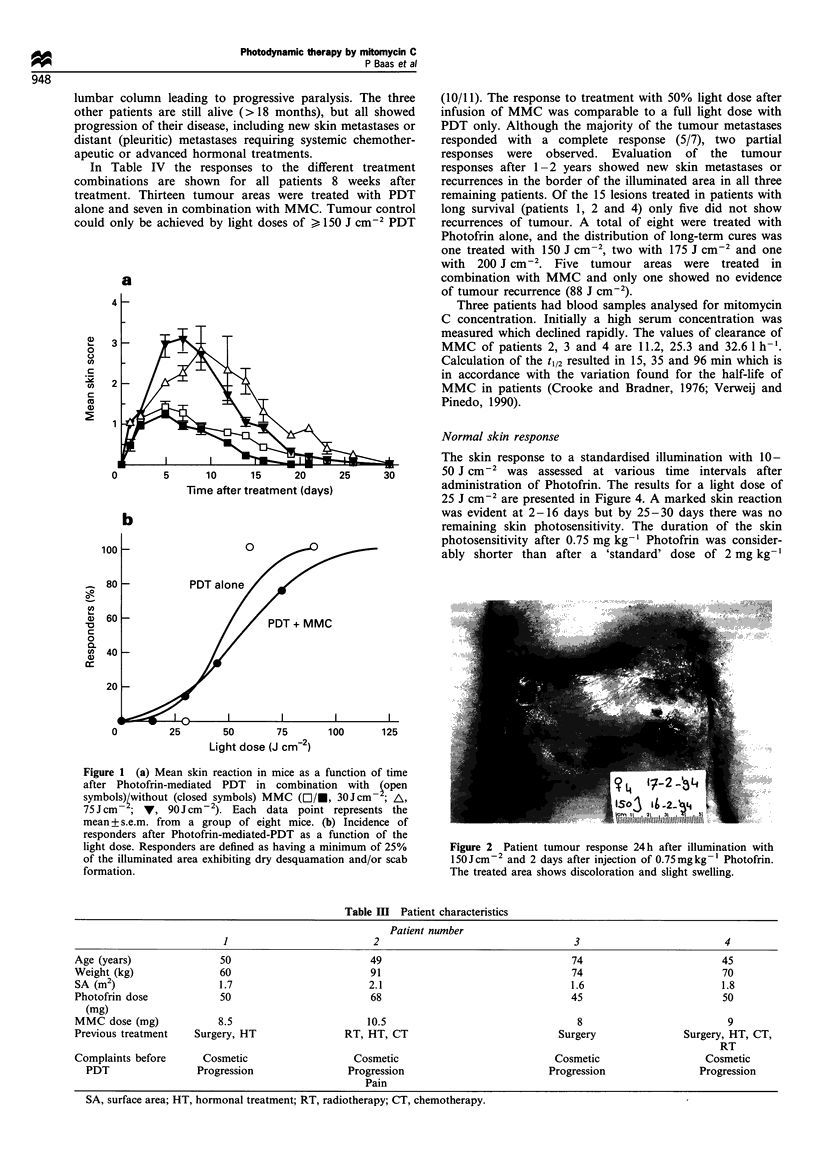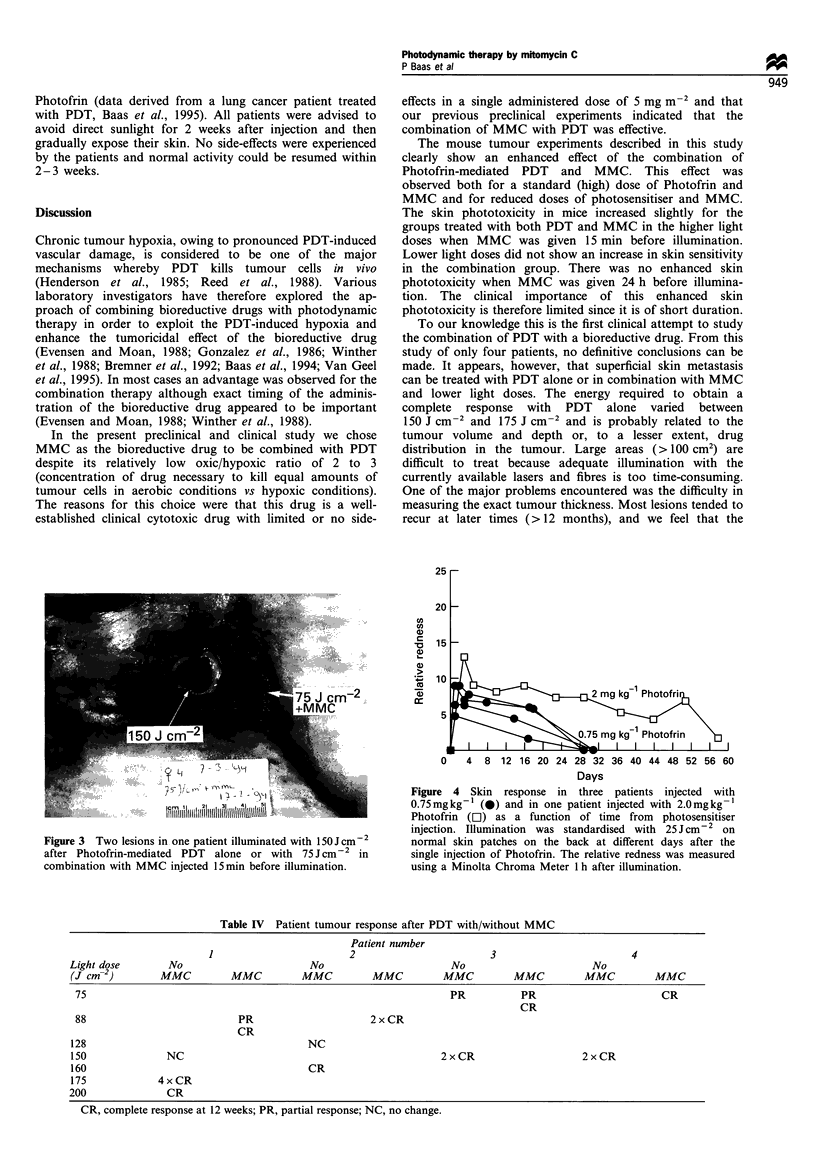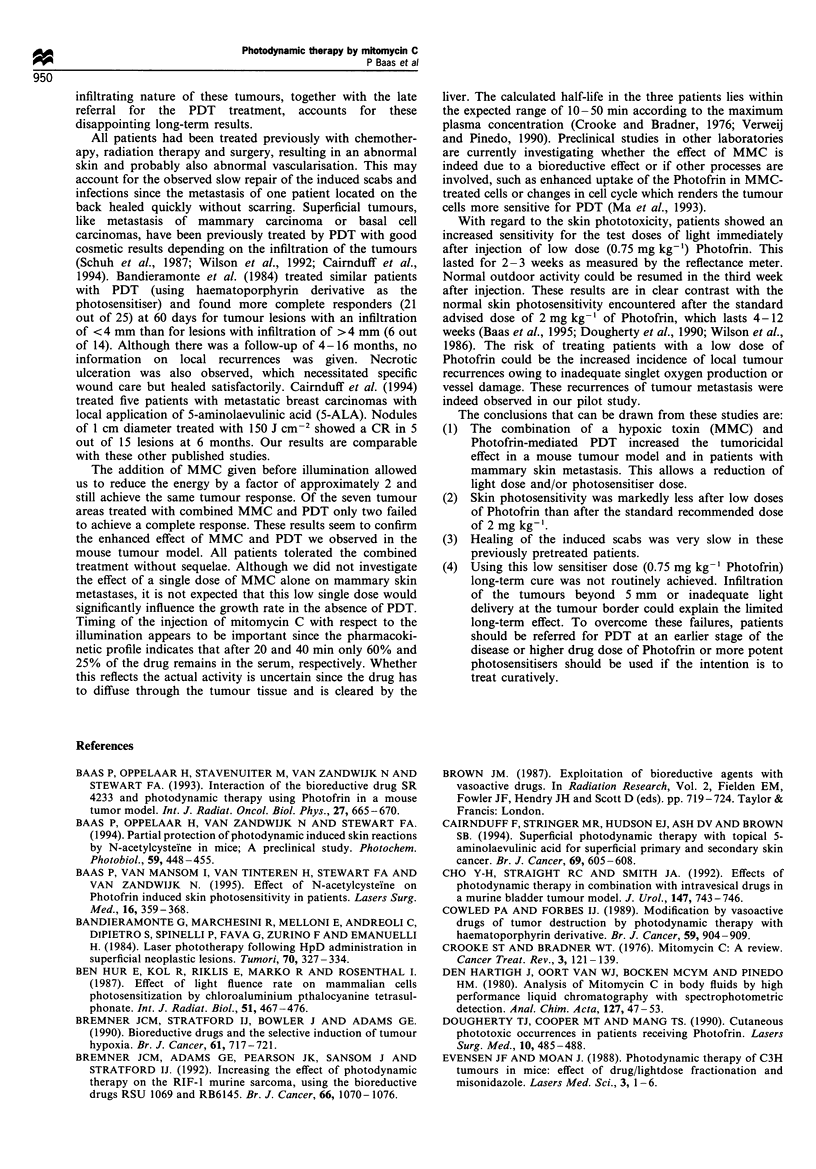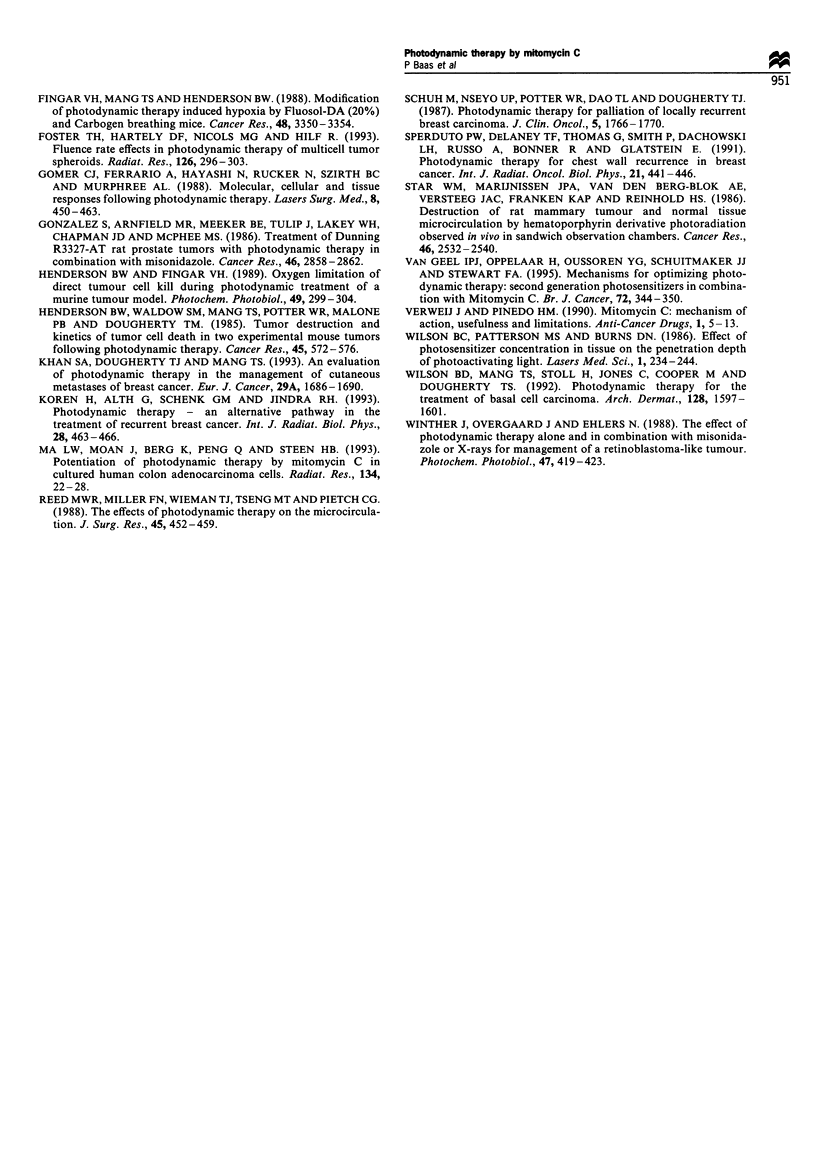# Enhancement of photodynamic therapy by mitomycin C: a preclinical and clinical study.

**DOI:** 10.1038/bjc.1996.186

**Published:** 1996-04

**Authors:** P. Baas, I. P. van Geel, H. Oppelaar, M. Meyer, J. H. Beynen, N. van Zandwijk, F. A. Stewart

**Affiliations:** Division of Medical Oncology, The Netherlands Cancer Institute, Antoni van Leeuwenhoek Hospital, Amsterdam, The Netherlands.

## Abstract

**Images:**


British Journal of Cancer (1996) 73, 945-951
© 1996 Stockton Press All rights reserved 0007-0920/96 $12.00

### Enhancement of photodynamic therapy by mitomycin C: a preclinical and clinical study

P Baas[1,4], IPJ van Geel[2], H Oppelaar[2], M Meyer[1], JH Beynen[3], N van Zandwijk[1] and FA Stewart[2]

*Divisions of [1]Medical Oncology, [2]Experimental Therapy, The Netherlands Cancer Institute/Antoni van Leeuwenhoek Hospital, Plesmanlaan 121, 1066 CX, Amsterdam, The Netherlands; [3]Department of Pharmacy, Slotervaart Hospital, Louwesweg 6, Amsterdam, The Netherlands.*

**Summary** Photodynamic therapy (PDT) using Photofrin was used in combination with a hypoxic toxin (mitomycin C, MMC) to treat four patients with recurrent skin metastasis of a mammary carcinoma. In preclinical experiments an additive effect was found for the combination of MMC and PDT for treating subcutaneous RIF1 tumours in mice. When interstitial PDT was combined with a low dose of MMC (administered 15 min before illumination), the Photofrin dose or light dose could be reduced by a factor of 2 in order to obtain equivalent cure rate or growth delay. In the clinical pilot study, a low dose of Photfrin (0.75 mg $kg^{-1}$) was used for PDT alone (superficial illumination) or combined with low-dose MMC (5 mg $m^{-2}$). Different tumour areas were illuminated with or without a preceding infusion of MMC. Both tumour response and skin photosensitivity were scored. After 8–12 weeks of treatment, tumour cure could be achieved by administering light doses $\geqslant$150 J $cm^{-2}$ for PDT alone and similar effects were obtained when light doses of 75–87.5 J $cm^{-2}$ were given after infusion with MMC. In all cases necrotic tissue of both tumour and surrounding skin was observed, which lasted for a mean of 5 months (range 2-20 months). Skin phototoxicity, tested by using a standardised illumination of skin patches on the back, lasted maximally 3 weeks. Three main conclusions could be drawn from these studies: (1) The enhanced effects of the combination of PDT and MMC observed in mouse tumours can be extrapolated to patients with mammary skin metastasis. (2) The combination of PDT and hypoxic toxins facilitates treatment by permitting lower doses of photosensitiser to be used (thereby reducing skin phototoxicity) or lower light doses (thereby reducing illumination times and allowing the possibility to treat larger tumour areas). (3) Restoration of skin after PDT in previously treated tumour areas (chemotherapy, radiation therapy and surgery) is very slow.

**Keywords:** photodynamic therapy; mitomycin C; bioreductive drugs; mammary carcinoma

Photodynamic therapy is now becoming a more accepted form of treatment for small superficial tumours. Its major advantage is the relatively selective treatment of tumours with preservation of the surrounding normal tissue. Preferential retention of the photosensitiser by the tumour tissue and selective illumination are the cornerstones in this treatment. To obtain total tumour eradication, the light used to excite the photosensitiser must penetrate the full depth of the tumour tissue (Ben Hur *et al.*, 1987) and sufficient photosensitiser and oxygen must be available for the formation of highly reactive singlet oxygen. Direct tumour cell kill may arise from this process but vasculature effects will also lead to hypoxia. If the hypoxia is deep and long lasting enough, additional tumour cells will be killed as a consequence of this hypoxia (Henderson *et al.*, 1985; Star *et al.*, 1986). Subcurative treatment, leading to recurrence of tumour can therefore be the result of inadequate light penetration, a lack of oxygen or low sensitiser dose. Tumour tissue is often not optimally vascularised and areas of hypoxia have been observed by many investigators, not only in animal models but also in human tumour tissues. The photochemical reaction itself will also consume oxygen and this can lead to an oxygen deficit during prolonged illumination, even when the vasculature is intact (Foster *et al.*, 1993). It is therefore believed that an important limitation in successful PDT is the lack of oxygen. Several studies have been directed to overcome this problem by techniques that can replace or increase the oxygen content in the tumour, e.g. addition of carbogen (Fingar *et al.*, 1988) or by increasing tumour blood flow (Foster *et al.*, 1993; Cowled and Forbes, 1989). An alternative approach is to exploit the PDT-induced hypoxia by the addition of a hypoxic toxin which will specifically target cells at low oxygen tensions (Baas *et al.*, 1993; Bremner *et al.*, 1990, 1992; Cho *et al.*, 1992; Gonzalez *et al.*, 1986).

Breast cancer is the most common cancer in females in all industrialised countries. Although the initial treatment of breast cancer is very successful, many patients are at risk of developing a recurrence in the course of their disease. Distant metastasis will be found in approximately half of the patients and 5–10% will suffer from local residual or recurrent disease. Local recurrences are treated with various modalities, such as surgery, hormonal or chemotherapy or radiation therapy. In spite of these treatments, some patients will still suffer from residual disease and complain initially of the cosmetic changes. If left untreated, ulceration and pain will become a serious risk. For these patients, photodynamic therapy could be an alternative treatment and it has been shown to be feasible by Kahn *et al.* (1993), Schuh *et al.* (1987) and Sperduto *et al.* (1991).

The major side-effect of PDT using Photofrin is skin phototoxicity since the injected photosensitiser is also retained by the skin. Sensitivity for intense light can last from 8 to 12 weeks after standard Photofrin doses of 2 mg $kg^{-1}$. A reduction in the duration of skin phototoxicity can be obtained by decreasing the photosensitiser dose (Wilson *et al.*, 1992). However, since the photochemical process is then less efficient, illumination times have to be increased (Kahn *et al.*, 1993). For patients with multiple lesions or large treatment areas, prolonged illumination times are undesirable. A combination of a hypoxic toxin and PDT could enable the use of a lower photosensitiser dose and/or reduced illumination times. We therefore initiated a study to determine the influence of a hypoxic toxin (mitomycin C) on the photodynamic effect in tumours and normal tissue of mice and a pilot study in patients with mammary skin metastases. The tumour response and the duration of the skin phototoxicity in patients was tested for a low dose of Photofrin. The pharmacokinetic profile of a single injection of mitomycin C was also measured.

Correspondence: P Baas
Received 7 August 1995; revised 17 November 1995; accepted 28 November 1995

### Materials and methods

### Preclinical experiments

All experiments were carried out in accordance with protocols approved by the local animal welfare committee and conformed to national and international laws. C3H/Km female mice (25–30 g) were inoculated with $1 \times 10^5$ RIF1 cells on the lower dorsum. Within 14 days most tumours had grown to a size of 5–6 mm diameter and were used for treatment. Tumour response was evaluated by measuring the tumour three times a week with vernier callipers and calculating the time to increase by 2 mm in mean diameter from the time of treatment (regrowth time). Cures were defined as no visible and palpable tumour 90 days after treatment. Cures were excluded from the analysis of mean regrowth times and were analysed separately. A minimum of six mice per dose group were treated.

Normal skin damage was assessed in female Balb/c mice (21–30 g at 14–30 weeks). The mice were anaesthetised with intraperitoneally (i.p.) Nembutal (sodium-pentobarbital 60 mg $kg^{-1}$; Abbot, The Netherlands) before hair on the back was plucked (area of approximately 5 $cm^2$). Photofrin was injected i.p. in a dose of 10 mg $kg^{-1}$ and the skin was illuminated superficially after 1 day. Skin response was measured three times weekly using a visual scoring scale [ranging from slight redness (grade 1), intense redness (grade 2), desquamation (grade 3) to scab formation (grade 4)] by two independent observers (Baas *et al.*, 1994). A minimum of eight mice per group were treated.

### Photosensitiser

Photofrin (QLT, Vancouver, Canada) was used in all experiments. Vials containing dry powder (15 mg) were diluted in 5% dextran to a concentration of 2 mg $kg^{-1}$. The drug was injected i.p. in a dose of 5 or 10 mg $kg^{-1}$ 24–30 h before illumination. The mice were housed in a darkened room for 2 weeks after injection of Photofrin.

### Mitomycin C

MMC (2 mg vials, Kyowa, Japan) was dissolved in sterile water to a concentration of 5 mg $ml^{-1}$. The drug was prepared directly before use and injected i.p. in a concentration of 5 mg $kg^{-1}$ 15 min or 24 h before the start of illumination. This drug concentration had been shown to cause minimal acute toxicity (Baas *et al.*, 1994).

### Interstitial photodynamic tumour treatment

Laser light was obtained from an argon dye laser (Spectra Physics model 171, San Jose, CA, USA) which pumped a dye laser (Spectra Physics model 375) tuned at $628 \pm 3$ nm. The laser light was transported via polystyrene fibres with a 1-cm-long terminal cylindrical diffusing tip (Baas *et al.*, 1993). The diffusing tip was inserted through the centre of the tumour. To avoid hyperthermal effects, the light fluence rate was fixed at 100 mW $cm^{-1}$. The energy deposition varied from 100 to 400 J $cm^{-1}$.

### Superficial photodynamic treatment

Superficial illumination of normal skin of mice was given by a cold light lamp (type KL 1500, Scott, Mainz, Germany, emission spectrum 300–700 nm) fitted with a 150 W halogen lamp (Osram, The Netherlands). A fluence rate of 200 mW $cm^{-2}$ was used to deliver energies of 0–150 J $cm^{-2}$ to skin patches of 2.5 $cm^2$ in a maximum illumination time of 12.5 min as described previously (Baas *et al.*, 1994). The maximum skin temperature with this fluence rate was 41–43°C.

### Statistical analysis

Means and standard errors were calculated for tumour regrowth times for each group. Cured tumours were excluded from this analysis. Comparisons between groups were by means of Breslow statistics stratified for the light dose. This is a modified version of the Kruskal–Wallis (or generalised Wilcoxon) test and allows cures to be incorporated in the analysis as sensored observations. A *P*-value of $<0.05$ was considered significant. Cures were also analysed separately by probit analysis to determine the light dose giving a 50% cure rate ($TCD_{50}$).

### Clinical experiments

Patients entering this pilot study had to fulfil the following criteria: a cytological/histological proof of the mammary skin metastasis or a recent increase in tumour size; total treatment surface should not exceed 100 $cm^2$, ECOG (Eastern Cooperative Oncology Group) performance score $\leqslant 3$; no concurrent chemotherapy, radiation or hormonal therapy in the last 4 weeks before treatment; normal haematology, liver and renal function; no previous treatment with PDT or MMC; no porphyria. A written informed consent was obtained from each patient and the experiment was approved by the local medical ethical committee.

### Photosensitiser and mitomycin C

Photofrin was injected in a dose of 0.75 mg $kg^{-1}$ intravenously. MMC (5 mg $m^{-2}$) was given 20 min before illumination in a slow push injection. Patient serum was obtained from the contralateral cubital vein after injection. MMC concentration was determined by high-performance liquid chromatography (HPLC) (Den Hartigh *et al.*, 1980).

### Photodynamic therapy

Skin metastases with a diameter of $<3.0$ cm and estimated thickness of $<0.5$ cm were considered suitable for illumination. Laser light (630 nm) obtained from the argon dye laser was transmitted via a quartz fibre with a microlens tip (Quadra Logic Technology, Pearl River, NY, USA). The patient was treated in a supine position with a beam spot of 7 $cm^2$ (diameter 3 cm) aimed at the tumour area at a mean distance of 25–30 cm. The fluence rate at the tumour surface was calculated to be 150 mW $cm^{-2}$. Energies of 125–200 J $cm^{-2}$ were administered in 15–25 min for the treatment with Photofrin alone. On day 1 after injection of the photosensitiser a test dose of 150 J $cm^{-2}$ was given to a tumour area and on day 2 other tumour areas were treated with the same dose if a discoloration had occurred in the treated area or with an increased light dose of 175 or 200 J $cm^{-2}$ if no significant changes had occurred. On day 3, MMC was injected as described above. Twenty and 40 min after infusion of the MMC new tumour areas were treated with half the light dose (e.g. 75 or 87.5 J $cm^{-2}$). On day 3 or 4, additional treatments were given to the remaining tumour areas using a light dose of 150 J $cm^{-2}$ without MMC. Evaluation of the response was performed every 3 weeks for the first 12 weeks and then every 4 months by visual inspection and photography. If possible cytological evaluation was also performed.

### Skin phototoxicity

The extent of skin phototoxicity induced by this low dose of Photofrin was determined using the method described by Baas *et al.* (1995). Skin patches of 2.5 $cm^2$ on the normal skin of the back were illuminated weekly after administration of the photosensitiser. The energy applied to the skin patches varied from 10–50 J $cm^{-2}$ (fluence rate 150 mW $cm^{-2}$). After 1 h the induced erythema was measured using a Minolta Chroma Meter (type CR-200, Minolta Camera, Osaka, Japan) and by visual scoring.

### Results

### Preclinical results

Previous published results had demonstrated that Photofrin-mediated PDT was enhanced by MMC given before illumination when high doses of both photosensitiser (10 mg $kg^{-1}$) and MMC (5 mg $kg^{-1}$) were given (Baas *et al.*, 1994). New studies demonstrate that the enhanced effect is still seen using lower doses of either photosensitiser (5 mg $kg^{-1}$) or MMC (2.5 mg $kg^{-1}$). All the combined treatments gave longer tumour regrowth times (Table I) and more cures (Table II) than PDT alone or MMC alone ($P<0.0001$). Tumour regrowth times decreased significantly ($P<0.0001$) when PDT was given with low doses of Photofrin (5 mg $kg^{-1}$) instead of the standard Photofrin dose of 10 mg $kg^{-1}$. The maximum effect was obtained using high doses of both drugs but the regrowth times and $TCD_{50}$ values (light dose required for 50% tumour cure) for 2.5 and 5 mg $kg^{-1}$ MMC with the high photosensitiser dose were not significantly different.

The mouse skin phototoxicity scoring system as described by Baas *et al.* (1995) was used to evaluate the combination of Photofrin (10 mg $kg^{-1}$, given 1 day before illumination) and MMC (5 mg $kg^{-1}$, given 15 min before illumination). Using the visible scoring system it was possible to obtain reproducible profiles for skin damage. The mean skin reactions for different light doses given 1 day after injection of Photofrin with or without MMC are shown in Figure 1a. A slight increase in mean skin reaction was observed when PDT was combined with MMC given 15 min before illumination. A light dose of 75 J $cm^{-2}$ in combination with MMC produced as much skin damage as 90 J $cm^{-2}$ PDT alone. MMC given 24 h before illumination did not increase skin reactions relative to PDT alone (data not shown). In order to compare skin reactions quantitatively after PDT, a light dose response curve was constructed for the incidence of dry and moist desquamation. Only animals with at least 25% of the illuminated area developing desquamation were scored as 'responders'. Figure 1b illustrates the results of these calculations. This analysis demonstrated no significant difference in the incidence of desquamation response between PDT alone or in combination with MMC (light doses of $49.0\pm6.3$ and $57.0\pm7.9$ J $cm^{-2}$ gave a response in 50% of the mice).

### Clinical results

Four patients were entered in the pilot study in a 2 year period. The patient characteristics are presented in Table III. All had previous treatments for their recurrent mammary metastases. The major complaints at time of PDT were progression of the tumours and the resultant cosmetic appearance. All patients were treated according to the protocol, and three patients fulfilled all inclusion criteria, including positive cytology. The fourth patient experienced progression of the lesions but the repeated cytological examination was negative (insufficient material).

In all patients a predictable course of tumour reaction was observed: no direct change of the tumour during the light treatment but a brown-bluish discolouration which developed after 24 h (Figure 2). Over the next 10 days the lesion turned into a dry black scab which was present for at least 8 weeks. Areas treated with light doses of $\geqslant150$ J $cm^{-2}$ (PDT alone) or some areas treated with 75–88 J $cm^{-2}$ in combination with MMC, had scabs lasting 8–20 months (Figure 3). In most treated tumour areas, normal skin reappeared after the scab had fallen off. In patient 1, skin metastases were also located on the back and treated according to the protocol. The scabs resolved soonest in these illuminated areas with perfect healing of the skin. In 3 of the 19 lesions treated an exudative local infection developed, two could be treated locally but one required a course of oral antibiotics. One treatment session was complicated by a burning sensation 14 min after the start of the treatment (administered dose: 128 J $cm^{-2}$). Further treatment was delayed for 24 h and no change occurred in the treated area. Further illumination procedures were uneventful. One patient died 4 months after treatment owing to newly diagnosed bone metastasis in the

**Table I** The effect of laser illumination and drugs on tumour regrowth times (days) in mice

| *Treatment* | *Light dose (J $cm^{-1}$)* 0 | 100 | 200 | 300 | 400 |
|---|---|---|---|---|---|
| MMC (2.5 mg $kg^{-1}$) | $4.1\pm0.2$ | – | – | – | – |
| MMC (5 mg $kg^{-1}$) | $5.8\pm0.5$ | – | – | – | – |
| PDT[a] | – | $7.9\pm1.0$ | $12.3\pm1.3$ | $13.1\pm1.3$ | $15.0\pm1.3$ |
| PDT[a]+MMC (2.5 mg $kg^{-1}$) | – | $15.0\pm1.3$ | $13.9\pm1.3$ | $14.3\pm1.3$ | $24.5\pm2.2$ |
| PDT[a]+MMC (5 mg $kg^{-1}$) | – | $20.8\pm1.3$ | $27.7\pm6.7$ | $27.3\pm6.0$ | $25.8\pm3.5$ |
| PDT[b] | $4.8\pm0.4$ | $11.5\pm0.9$ | $16.0\pm0.9$ | $16.5\pm1.0$ | $16.0\pm0.8$ |
| PDT[b]+MMC (2.5 mg $kg^{-1}$) | – | – | $20.7\pm2.3$ | $23.1\pm2.8$ | $24.2\pm6.4$ |
| PDT[b]+MMC (5 mg $kg^{-1}$) | $8.5\pm0.8$ | $18.2\pm3.2$ | $23.1\pm1.0$ | $22.3\pm1.5$ | $31.9\pm0.9$ |

[a]5 mg $kg^{-1}$ Photofrin. [b]10 mg $kg^{-1}$ Photofrin/previously published by Baas *et al.* (1994).

**Table II** The effect of PDT+MMC on local tumour control in mice (number of cured tumours per dose group)

| *Light dose (J $cm^{-2}$)* | *PDT[a] alone* | *PDT[a]+MMC 2.5 mg $kg^{-1}$* | *PDT[a]+MMC 5 mg $kg^{-1}$* | *PDT[b]+MMC (5 mg $kg^{-1}$)* |
|---|---|---|---|---|
| 0 | 0/18 | 0/6 | 0/8 | – |
| 100 | 1/25 | – | 0/8 | 0/8 |
| 200 | 0/23 | 1/8 | 4/8 | 0/8 |
| 300 | 1/29 | 4/10 | 3/10 | 2/8 |
| 400 | 1/24 | 5/8 | 7/10 | 1/8 |
| 500 | 5/15 | – | – | – |
| $TCD_{50}$ | $731\pm170$ | $348\pm41$ | $319\pm49$ | ND |

[a]10 mg $kg^{-1}$ Photofrin. [b]5 mg $kg^{-1}$ Photofrin. ND, not determined.

lumbar column leading to progressive paralysis. The three other patients are still alive (>18 months), but all showed progression of their disease, including new skin metastases or distant (pleuritic) metastases requiring systemic chemotherapeutic or advanced hormonal treatments.

In Table IV the responses to the different treatment combinations are shown for all patients 8 weeks after treatment. Thirteen tumour areas were treated with PDT alone and seven in combination with MMC. Tumour control could only be achieved by light doses of $\geqslant 150$ J $cm^{-2}$ PDT (10/11). The response to treatment with 50% light dose after infusion of MMC was comparable to a full light dose with PDT only. Although the majority of the tumour metastases responded with a complete response (5/7), two partial responses were observed. Evaluation of the tumour responses after 1–2 years showed new skin metastases or recurrences in the border of the illuminated area in all three remaining patients. Of the 15 lesions treated in patients with long survival (patients 1, 2 and 4) only five did not show recurrences of tumour. A total of eight were treated with Photofrin alone, and the distribution of long-term cures was one treated with 150 J $cm^{-2}$, two with 175 J $cm^{-2}$ and one with 200 J $cm^{-2}$. Five tumour areas were treated in combination with MMC and only one showed no evidence of tumour recurrence (88 J $cm^{-2}$).

Three patients had blood samples analysed for mitomycin C concentration. Initially a high serum concentration was measured which declined rapidly. The values of clearance of MMC of patients 2, 3 and 4 are 11.2, 25.3 and 32.6 l $h^{-1}$. Calculation of the $t_{1/2}$ resulted in 15, 35 and 96 min which is in accordance with the variation found for the half-life of MMC in patients (Crooke and Bradner, 1976; Verweij and Pinedo, 1990).

*Normal skin response*

The skin response to a standardised illumination with 10–50 J $cm^{-2}$ was assessed at various time intervals after administration of Photofrin. The results for a light dose of 25 J $cm^{-2}$ are presented in Figure 4. A marked skin reaction was evident at 2–16 days but by 25–30 days there was no remaining skin photosensitivity. The duration of the skin photosensitivity after 0.75 mg $kg^{-1}$ Photofrin was considerably shorter than after a 'standard' dose of 2 mg $kg^{-1}$

**Figure 1** (**a**) Mean skin reaction in mice as a function of time after Photofrin-mediated PDT in combination with (open symbols)/without (closed symbols) MMC (□/■, 30 J $cm^{-2}$; △, 75 J $cm^{-2}$; ▼, 90 J $cm^{-2}$). Each data point represents the mean ± s.e.m. from a group of eight mice. (**b**) Incidence of responders after Photofrin-mediated-PDT as a function of the light dose. Responders are defined as having a minimum of 25% of the illuminated area exhibiting dry desquamation and/or scab formation.

**Figure 2** Patient tumour response 24 h after illumination with 150 J $cm^{-2}$ and 2 days after injection of 0.75 mg $kg^{-1}$ Photofrin. The treated area shows discoloration and slight swelling.

**Table III** Patient characteristics

| | Patient number | | | |
|---|---|---|---|---|
| | *1* | *2* | *3* | *4* |
| Age (years) | 50 | 49 | 74 | 45 |
| Weight (kg) | 60 | 91 | 74 | 70 |
| SA ($m^2$) | 1.7 | 2.1 | 1.6 | 1.8 |
| Photofrin dose (mg) | 50 | 68 | 45 | 50 |
| MMC dose (mg) | 8.5 | 10.5 | 8 | 9 |
| Previous treatment | Surgery, HT | RT, HT, CT | Surgery | Surgery, HT, CT, RT |
| Complaints before PDT | Cosmetic Progression | Cosmetic Progression Pain | Cosmetic Progression | Cosmetic Progression |

SA, surface area; HT, hormonal treatment; RT, radiotherapy; CT, chemotherapy.

Photofrin (data derived from a lung cancer patient treated with PDT, Baas *et al.*, 1995). All patients were advised to avoid direct sunlight for 2 weeks after injection and then gradually expose their skin. No side-effects were experienced by the patients and normal activity could be resumed within 2–3 weeks.

### Discussion

Chronic tumour hypoxia, owing to pronounced PDT-induced vascular damage, is considered to be one of the major mechanisms whereby PDT kills tumour cells *in vivo* (Henderson *et al.*, 1985; Reed *et al.*, 1988). Various laboratory investigators have therefore explored the approach of combining bioreductive drugs with photodynamic therapy in order to exploit the PDT-induced hypoxia and enhance the tumoricidal effect of the bioreductive drug (Evensen and Moan, 1988; Gonzalez *et al.*, 1986; Winther *et al.*, 1988; Bremner *et al.*, 1992; Baas *et al.*, 1994; Van Geel *et al.*, 1995). In most cases an advantage was observed for the combination therapy although exact timing of the administration of the bioreductive drug appeared to be important (Evensen and Moan, 1988; Winther *et al.*, 1988).

In the present preclinical and clinical study we chose MMC as the bioreductive drug to be combined with PDT despite its relatively low oxic/hypoxic ratio of 2 to 3 (concentration of drug necessary to kill equal amounts of tumour cells in aerobic conditions *vs* hypoxic conditions). The reasons for this choice were that this drug is a well-established clinical cytotoxic drug with limited or no side-effects in a single administered dose of 5 mg $m^{-2}$ and that our previous preclinical experiments indicated that the combination of MMC with PDT was effective.

The mouse tumour experiments described in this study clearly show an enhanced effect of the combination of Photofrin-mediated PDT and MMC. This effect was observed both for a standard (high) dose of Photofrin and MMC and for reduced doses of photosensitiser and MMC. The skin phototoxicity in mice increased slightly for the groups treated with both PDT and MMC in the higher light doses when MMC was given 15 min before illumination. Lower light doses did not show an increase in skin sensitivity in the combination group. There was no enhanced skin phototoxicity when MMC was given 24 h before illumination. The clinical importance of this enhanced skin phototoxicity is therefore limited since it is of short duration.

To our knowledge this is the first clinical attempt to study the combination of PDT with a bioreductive drug. From this study of only four patients, no definitive conclusions can be made. It appears, however, that superficial skin metastasis can be treated with PDT alone or in combination with MMC and lower light doses. The energy required to obtain a complete response with PDT alone varied between 150 J $cm^{-2}$ and 175 J $cm^{-2}$ and is probably related to the tumour volume and depth or, to a lesser extent, drug distribution in the tumour. Large areas (>100 $cm^2$) are difficult to treat because adequate illumination with the currently available lasers and fibres is too time-consuming. One of the major problems encountered was the difficulty in measuring the exact tumour thickness. Most lesions tended to recur at later times (>12 months), and we feel that the

**Figure 3** Two lesions in one patient illuminated with 150 J $cm^{-2}$ after Photofrin-mediated PDT alone or with 75 J $cm^{-2}$ in combination with MMC injected 15 min before illumination.

**Figure 4** Skin response in three patients injected with 0.75 mg $kg^{-1}$ (●) and in one patient injected with 2.0 mg $kg^{-1}$ Photofrin (□) as a function of time from photosensitiser injection. Illumination was standardised with 25 J $cm^{-2}$ on normal skin patches on the back at different days after the single injection of Photofrin. The relative redness was measured using a Minolta Chroma Meter 1 h after illumination.

**Table IV** Patient tumour response after PDT with/without MMC

| | *Patient number* 1 | | 2 | | 3 | | 4 | |
|---|---|---|---|---|---|---|---|---|
| *Light dose ($J\ cm^{-2}$)* | *No MMC* | *MMC* | *No MMC* | *MMC* | *No MMC* | *MMC* | *No MMC* | *MMC* |
| 75 | | | | | PR | PR<br>CR | | CR |
| 88 | | PR<br>CR | | 2×CR | | | | |
| 128 | | | NC | | | | | |
| 150 | NC | | | | 2×CR | | 2×CR | |
| 160 | | | CR | | | | | |
| 175 | 4×CR | | | | | | | |
| 200 | CR | | | | | | | |

CR, complete response at 12 weeks; PR, partial response; NC, no change.

infiltrating nature of these tumours, together with the late referral for the PDT treatment, accounts for these disappointing long-term results.

All patients had been treated previously with chemotherapy, radiation therapy and surgery, resulting in an abnormal skin and probably also abnormal vascularisation. This may account for the observed slow repair of the induced scabs and infections since the metastasis of one patient located on the back healed quickly without scarring. Superficial tumours, like metastasis of mammary carcinoma or basal cell carcinomas, have been previously treated by PDT with good cosmetic results depending on the infiltration of the tumours (Schuh *et al.*, 1987; Wilson *et al.*, 1992; Cairnduff *et al.*, 1994). Bandieramonte *et al.* (1984) treated similar patients with PDT (using haematoporphyrin derivative as the photosensitiser) and found more complete responders (21 out of 25) at 60 days for tumour lesions with an infiltration of <4 mm than for lesions with infiltration of >4 mm (6 out of 14). Although there was a follow-up of 4–16 months, no information on local recurrences was given. Necrotic ulceration was also observed, which necessitated specific wound care but healed satisfactorily. Cairnduff *et al.* (1994) treated five patients with metastatic breast carcinomas with local application of 5-aminolaevulinic acid (5-ALA). Nodules of 1 cm diameter treated with 150 J $cm^{-2}$ showed a CR in 5 out of 15 lesions at 6 months. Our results are comparable with these other published studies.

The addition of MMC given before illumination allowed us to reduce the energy by a factor of approximately 2 and still achieve the same tumour response. Of the seven tumour areas treated with combined MMC and PDT only two failed to achieve a complete response. These results seem to confirm the enhanced effect of MMC and PDT we observed in the mouse tumour model. All patients tolerated the combined treatment without sequelae. Although we did not investigate the effect of a single dose of MMC alone on mammary skin metastases, it is not expected that this low single dose would significantly influence the growth rate in the absence of PDT. Timing of the injection of mitomycin C with respect to the illumination appears to be important since the pharmacokinetic profile indicates that after 20 and 40 min only 60% and 25% of the drug remains in the serum, respectively. Whether this reflects the actual activity is uncertain since the drug has to diffuse through the tumour tissue and is cleared by the liver. The calculated half-life in the three patients lies within the expected range of 10–50 min according to the maximum plasma concentration (Crooke and Bradner, 1976; Verweij and Pinedo, 1990). Preclinical studies in other laboratories are currently investigating whether the effect of MMC is indeed due to a bioreductive effect or if other processes are involved, such as enhanced uptake of the Photofrin in MMC-treated cells or changes in cell cycle which renders the tumour cells more sensitive for PDT (Ma *et al.*, 1993).

With regard to the skin phototoxicity, patients showed an increased sensitivity for the test doses of light immediately after injection of low dose (0.75 mg $kg^{-1}$) Photofrin. This lasted for 2–3 weeks as measured by the reflectance meter. Normal outdoor activity could be resumed in the third week after injection. These results are in clear contrast with the normal skin photosensitivity encountered after the standard advised dose of 2 mg $kg^{-1}$ of Photofrin, which lasts 4–12 weeks (Baas *et al.*, 1995; Dougherty *et al.*, 1990; Wilson *et al.*, 1986). The risk of treating patients with a low dose of Photofrin could be the increased incidence of local tumour recurrences owing to inadequate singlet oxygen production or vessel damage. These recurrences of tumour metastasis were indeed observed in our pilot study.

The conclusions that can be drawn from these studies are:

(1) The combination of a hypoxic toxin (MMC) and Photofrin-mediated PDT increased the tumoricidal effect in a mouse tumour model and in patients with mammary skin metastasis. This allows a reduction of light dose and/or photosensitiser dose.
(2) Skin photosensitivity was markedly less after low doses of Photofrin than after the standard recommended dose of 2 mg $kg^{-1}$.
(3) Healing of the induced scabs was very slow in these previously pretreated patients.
(4) Using this low sensitiser dose (0.75 mg $kg^{-1}$ Photofrin) long-term cure was not routinely achieved. Infiltration of the tumours beyond 5 mm or inadequate light delivery at the tumour border could explain the limited long-term effect. To overcome these failures, patients should be referred for PDT at an earlier stage of the disease or higher drug dose of Photofrin or more potent photosensitisers should be used if the intention is to treat curatively.

### References

BAAS P, OPPELAAR H, STAVENUITER M, VAN ZANDWIJK N AND STEWART FA. (1993). Interaction of the bioreductive drug SR 4233 and photodynamic therapy using Photofrin in a mouse tumor model. *Int. J. Radiat. Oncol. Biol. Phys.*, **27**, 665–670.

BAAS P, OPPELAAR H, VAN ZANDWIJK N AND STEWART FA. (1994). Partial protection of photodynamic induced skin reactions by N-acetylcysteïne in mice; A preclinical study. *Photochem. Photobiol.*, **59**, 448–455.

BAAS P, VAN MANSOM I, VAN TINTEREN H, STEWART FA AND VAN ZANDWIJK N. (1995). Effect of N-acetylcysteïne on Photofrin induced skin photosensitivity in patients. *Lasers Surg. Med.*, **16**, 359–368.

BANDIERAMONTE G, MARCHESINI R, MELLONI E, ANDREOLI C, DIPIETRO S, SPINELLI P, FAVA G, ZURINO F AND EMANUELLI H. (1984). Laser phototherapy following HpD administration in superficial neoplastic lesions. *Tumori*, **70**, 327–334.

BEN HUR E, KOL R, RIKLIS E, MARKO R AND ROSENTHAL I. (1987). Effect of light fluence rate on mammalian cells photosensitization by chloroaluminium pthalocyanine tetrasulphonate. *Int. J. Radiat. Biol.*, **51**, 467–476.

BREMNER JCM, STRATFORD IJ, BOWLER J AND ADAMS GE. (1990). Bioreductive drugs and the selective induction of tumour hypoxia. *Br. J. Cancer*, **61**, 717–721.

BREMNER JCM, ADAMS GE, PEARSON JK, SANSOM J AND STRATFORD IJ. (1992). Increasing the effect of photodynamic therapy on the RIF-1 murine sarcoma, using the bioreductive drugs RSU 1069 and RB6145. *Br. J. Cancer*, **66**, 1070–1076.

BROWN JM. (1987). Exploitation of bioreductive agents with vasoactive drugs. In *Radiation Research*, Vol. 2, Fielden EM, Fowler JF, Hendry JH and Scott D (eds). pp. 719–724. Taylor & Francis: London.

CAIRNDUFF F, STRINGER MR, HUDSON EJ, ASH DV AND BROWN SB. (1994). Superficial photodynamic therapy with topical 5-aminolaevulinic acid for superficial primary and secondary skin cancer. *Br. J. Cancer*, **69**, 605–608.

CHO Y-H, STRAIGHT RC AND SMITH JA. (1992). Effects of photodynamic therapy in combination with intravesical drugs in a murine bladder tumour model. *J. Urol.*, **147**, 743–746.

COWLED PA AND FORBES IJ. (1989). Modification by vasoactive drugs of tumor destruction by photodynamic therapy with haematoporphyrin derivative. *Br. J. Cancer*, **59**, 904–909.

CROOKE ST AND BRADNER WT. (1976). Mitomycin C: A review. *Cancer Treat. Rev.*, **3**, 121–139.

DEN HARTIGH J, OORT VAN WJ, BOCKEN MCYM AND PINEDO HM. (1980). Analysis of Mitomycin C in body fluids by high performance liquid chromatography with spectrophotometric detection. *Anal. Chim. Acta*, **127**, 47–53.

DOUGHERTY TJ, COOPER MT AND MANG TS. (1990). Cutaneous phototoxic occurrences in patients receiving Photofrin. *Lasers Surg. Med.*, **10**, 485–488.

EVENSEN JF AND MOAN J. (1988). Photodynamic therapy of C3H tumours in mice: effect of drug/lightdose fractionation and misonidazole. *Lasers Med. Sci.*, **3**, 1–6.

FINGAR VH, MANG TS AND HENDERSON BW. (1988). Modification of photodynamic therapy induced hypoxia by Fluosol-DA (20%) and Carbogen breathing mice. *Cancer Res.*, **48,** 3350–3354.

FOSTER TH, HARTELY DF, NICOLS MG AND HILF R. (1993). Fluence rate effects in photodynamic therapy of multicell tumor spheroids. *Radiat. Res.*, **126,** 296–303.

GOMER CJ, FERRARIO A, HAYASHI N, RUCKER N, SZIRTH BC AND MURPHREE AL. (1988). Molecular, cellular and tissue responses following photodynamic therapy. *Lasers Surg. Med.*, **8,** 450–463.

GONZALEZ S, ARNFIELD MR, MEEKER BE, TULIP J, LAKEY WH, CHAPMAN JD AND MCPHEE MS. (1986). Treatment of Dunning R3327-AT rat prostate tumors with photodynamic therapy in combination with misonidazole. *Cancer Res.*, **46,** 2858–2862.

HENDERSON BW AND FINGAR VH. (1989). Oxygen limitation of direct tumour cell kill during photodynamic treatment of a murine tumour model. *Photochem. Photobiol.*, **49,** 299–304.

HENDERSON BW, WALDOW SM, MANG TS, POTTER WR, MALONE PB AND DOUGHERTY TM. (1985). Tumor destruction and kinetics of tumor cell death in two experimental mouse tumors following photodynamic therapy. *Cancer Res.*, **45,** 572–576.

KHAN SA, DOUGHERTY TJ AND MANG TS. (1993). An evaluation of photodynamic therapy in the management of cutaneous metastases of breast cancer. *Eur. J. Cancer*, **29A,** 1686–1690.

KOREN H, ALTH G, SCHENK GM AND JINDRA RH. (1993). Photodynamic therapy – an alternative pathway in the treatment of recurrent breast cancer. *Int. J. Radiat. Biol. Phys.*, **28,** 463–466.

MA LW, MOAN J, BERG K, PENG Q AND STEEN HB. (1993). Potentiation of photodynamic therapy by mitomycin C in cultured human colon adenocarcinoma cells. *Radiat. Res.*, **134,** 22–28.

REED MWR, MILLER FN, WIEMAN TJ, TSENG MT AND PIETCH CG. (1988). The effects of photodynamic therapy on the microcirculation. *J. Surg. Res.*, **45,** 452–459.

SCHUH M, NSEYO UP, POTTER WR, DAO TL AND DOUGHERTY TJ. (1987). Photodynamic therapy for palliation of locally recurrent breast carcinoma. *J. Clin. Oncol.*, **5,** 1766–1770.

SPERDUTO PW, DELANEY TF, THOMAS G, SMITH P, DACHOWSKI LH, RUSSO A, BONNER R AND GLATSTEIN E. (1991). Photodynamic therapy for chest wall recurrence in breast cancer. *Int. J. Radiat. Oncol. Biol. Phys.*, **21,** 441–446.

STAR WM, MARIJNISSEN JPA, VAN DEN BERG-BLOK AE, VERSTEEG JAC, FRANKEN KAP AND REINHOLD HS. (1986). Destruction of rat mammary tumour and normal tissue microcirculation by hematoporphyrin derivative photoradiation observed *in vivo* in sandwich observation chambers. *Cancer Res.*, **46,** 2532–2540.

VAN GEEL IPJ, OPPELAAR H, OUSSOREN YG, SCHUITMAKER JJ AND STEWART FA. (1995). Mechanisms for optimizing photodynamic therapy: second generation photosensitizers in combination with Mitomycin C. *Br. J. Cancer*, **72,** 344–350.

VERWEIJ J AND PINEDO HM. (1990). Mitomycin C: mechanism of action, usefulness and limitations. *Anti-Cancer Drugs*, **1,** 5–13.

WILSON BC, PATTERSON MS AND BURNS DN. (1986). Effect of photosensitizer concentration in tissue on the penetration depth of photoactivating light. *Lasers Med. Sci.*, **1,** 234–244.

WILSON BD, MANG TS, STOLL H, JONES C, COOPER M AND DOUGHERTY TS. (1992). Photodynamic therapy for the treatment of basal cell carcinoma. *Arch. Dermat.*, **128,** 1597–1601.

WINTHER J, OVERGAARD J AND EHLERS N. (1988). The effect of photodynamic therapy alone and in combination with misonidazole or X-rays for management of a retinoblastoma-like tumour. *Photochem. Photobiol.*, **47,** 419–423.